# Supplementary material for: Antibiotic Prescribing Quality in Out-of-Hours Primary Care and Critical Appraisal of Disease-Specific Quality Indicators
Source: Antibiotics (Basel). 2019 Jun 12;8(2):79. doi: 10.3390/antibiotics8020079 (PMC6628021; doi:10.3390/antibiotics8020079)
Supplement: Supplementary file 1 [file antibiotics-08-00079-s001.zip › Table S1 2004-2009 vs 2016-2018.docx]

Table S1: Average value (%) for the disease-specific antibiotic prescribing quality indicators over a six-year period from 1 January 2004 to 31 December 2009 during out-of-hours vs value (%) for the disease-specific antibiotic prescribing quality indicators over a two-year period from 1 July 2016 to 30 June 2018

| ICPC-Code | Label | 2004-2009 | 2016-2018 | 2004-2009 | 2016-2018 | 2004-2009 | 2016-2018 |
| --- | --- | --- | --- | --- | --- | --- | --- |
|  |  | A (<20%) | | B (>80%) | | C (<5%) | |
| H71 | Acute otitis media | 62 | 64 [53-72] | 42 | 74 [72-81] | < 1 | 1 [0-1] |
| R74 | Acute upper respiratory tract infection | 32 | 30 [15-41] | 2 | 3 [2-7] | 2 | 2 [0-2] |
| R75 | Acute sinusitis | 65 | 51 [39-57] | 23 | 40 [35-51] | 4 | 7 [2-9] |
| R76 | Acute tonsillitis | 76 | 77 [65-87] | 11 | 6 [3-15] | < 1 | 1 [0-1] |
|  |  |  | A (<30%) |  | B (>80%) |  | C (<5%) |
| R78 | Acute bronchitis | 73 | 69 [58-75] | 34 | 42 [37-54] | 7 | 11 [5-15] |
|  |  |  | A (>80%) |  | B (>80%) |  | C (<5%) |
| R81 | Pneumonia | 72 | 80 [70-85] | 44 | 46 [42-62] | 10 | 15 [2-20] |
| U71 | Acute cystitis | 96 | 91 [77-95] | 40 | 69 [64-77] | 52 | 25 [18-32] |
