# Supplementary material for: Antibiotic Prescribing Quality in Out-of-Hours Primary Care and Critical Appraisal of Disease-Specific Quality Indicators
Source: Antibiotics (Basel). 2019 Jun 12;8(2):79. doi: 10.3390/antibiotics8020079 (PMC6628021; doi:10.3390/antibiotics8020079)
Supplement: Supplementary file 1 [file antibiotics-08-00079-s001.zip › Table S2.docx]

**Table S2.** Number of antibiotic prescriptions in a 2 year time frame linked to symptom diagnosis* (total antibiotic prescriptions = 26 436)

| **ICPC-code** | **Label** | **Number of antibiotic prescriptions** | **Percentage on total** |
| --- | --- | --- | --- |
| R05 | Cough | 260 | 0,98% |
| R21 | Throat symptom/complaint | 104 | 0,39% |
| A02 | Chills | 101 | 0,38% |
| H01 | Ear pain/earache | 37 | 0,14% |
| D11 | Diarrhoea | 36 | 0,14% |
| D01 | Abdominal pain/cramps general | 29 | 0,11% |
| H04 | Ear discharge | 29 | 0,11% |
| Other symptom diagnoses (each less than 0.1% of total) |  | 458 | 1.73% |
| **TOTAL** |  | **996** | **3.77 %** |

*symptom code according to the International Classification of Primary Care – 2nd Edition (ICPC-2-R) i.e. in each chapter code ending with 01 till 29 except S03, S09-S11
