# Supplementary material for: Antibiotic Prescribing Quality in Out-of-Hours Primary Care and Critical Appraisal of Disease-Specific Quality Indicators
Source: Antibiotics (Basel). 2019 Jun 12;8(2):79. doi: 10.3390/antibiotics8020079 (PMC6628021; doi:10.3390/antibiotics8020079)
Supplement: Supplementary file 1 [file antibiotics-08-00079-s001.zip › Table S3&S4.docx]

**Table S3.** Number of antibiotic prescriptions in a 2 year time frame linked to ICPC code R74

| **ATC code** | **substance name** | **number of prescriptions** | **percentage** |
| --- | --- | --- | --- |
| J01CA04 | amoxicillin | 2319 | 63% |
| J01CR02 | amoxicillin and beta-lactamase inhibitor | 423 | 12% |
| J01FA10 | azithromycin | 364 | 10% |
| J01FA09 | clarithromycin | 143 | 4% |
| J01CE02 | phenoxymethylpenicillin | 92 | 3% |
| J01DB05 | cefadroxil | 79 | 2% |
| J01MA14 | moxifloxacin | 71 | 2% |
| J01BA52 | thiamphenicol, combinations | 68 | 2% |
| J01DC02 | cefuroxime | 61 | 2% |
| Other* |  | 46 | 1% |
| **TOTAL** |  | **3666** |  |

*less than 1% each

**Table S4.** Number of antibiotic prescriptions in a 2 year time frame linked to ICPC code R76

| **ATC code** | **substance name** | **number of prescriptions** | **percentage** |
| --- | --- | --- | --- |
| J01CA04 | amoxicillin | 1541 | 66% |
| J01CR02 | amoxicillin and beta-lactamase inhibitor | 316 | 14% |
| J01CE02 | phenoxymethylpenicillin | 144 | 6% |
| J01FA10 | azithromycin | 100 | 4% |
| J01DB05 | cefadroxil | 92 | 4% |
| J01FA09 | clarithromycin | 73 | 3% |
| J01DC02 | cefuroxime | 25 | 1% |
| J01FF01 | clindamycin | 12 | 1% |
| J01MA14 | moxifloxacin | 12 | 1% |
| Other* |  | 20 | 1% |
| **TOTAL** |  | **2335** |  |

*less than 1% each
